# Are School‐Aged Children Who Self‐Identify as Neurodivergent at Greater Risk of Substance Misuse? A Study of 20 000 Secondary School Students

**DOI:** 10.1111/cch.70357

**Published:** 2026-09-24

**Authors:** John Headley Ward, Francesca Happé, Mina Fazel

**Affiliations:** ^1^ Department of Psychiatry University of Oxford Oxford UK; ^2^ Department of Psychiatry University of Cambridge Cambridge UK; ^3^ Cambridgeshire and Peterborough NHS Foundation Trust Cambridge UK; ^4^ Social, Genetic and Developmental Psychiatry Centre, Institute of Psychiatry, Psychology and Neuroscience King's College London London UK

**Keywords:** ADHD, adolescents, autism, neurodiversity, substance use

## Abstract

**Background:**

Neurodiversity is becoming a more widely used term amongst the general population, recognising neurodivergent people outside of traditional deficit‐based models of neurodevelopmental conditions such as autism, ADHD, dyslexia and dyspraxia. Although neurodevelopmental conditions have been associated with a variety of externalising behaviours, including substance misuse, there is limited research looking at whether the broader neurodivergent population may also share these risks. This study's primary aim was to understand the relationship between self‐reported neurodivergence and different forms of substance misuse in adolescents.

**Methods:**

This study uses data from the 2023 wave of the OxWell Student Survey, analysing responses from 21 749 secondary school‐aged children in England. Firth logistic regression was used to analyse the relationship between self‐reported neurodivergence and frequent use of substances (vapes, cigarettes, alcohol and recreational drugs). Models were adjusted for demographic variables (gender, age, ethnicity, socioeconomic status and family history of substance use) and psychological variables (well‐being scores, anxiety/depression measures).

**Results:**

25.1% of our sample identified as neurodivergent. Our analyses demonstrate that although levels of frequent substance misuse are generally low amongst school‐aged children (< 10%), neurodivergent children were significantly more likely to be in higher substance use categories (daily vaping/smoking, frequent drinking, binge drinking and lifetime illicit substance use).

**Conclusions:**

Our findings provide preliminary evidence that children who identify as neurodivergent are at greater risk of substance misuse, even after adjusting for other known risk factors. Furthermore, this study supports the need for scalable school‐based substance use preventive interventions that are applicable for neurodivergent young people.

## Summary

- Many young people now self‐identify as neurodivergent, incorporating a larger proportion of adolescents than those who have diagnosed neurodevelopmental conditions (e.g., autism and ADHD).
- Neurodivergent adolescents are at significantly higher risk of frequent substance use than their neurotypical peers, including vaping, smoking, binge drinking and illicit drug use. These differences persist even after adjusting for demographic and psychological risk factors
- These adolescents may have unmet needs around substance misuse, and it is unclear what preventive and intervention support could best be provided to them. The support we looked at—Education, Health and Care Plans (EHCPs)—had no significant effect on the relationship between neurodivergence and substance use.
- The concept of self‐medication, possibly for symptoms of anxiety or low mood, emerged as a possible explanation for substance use in some neurodivergent students, especially those reporting mental health‐related motivations for vaping.
- The findings support the importance of ensuring that any interventions for substance misuse in school‐aged populations consider the needs of neurodivergent students.

**Abbreviations**
ADHD: attention deficit hyperactivity disorder
ASD: autism spectrum disorders
EHCP: Education, Health and Care Plan
IMD: Index of Multiple Deprivation
ND: neurodivergent
NT: neurotypical
PPI: patient and public involvement
RCADS: Revised Children's Anxiety and Depression Scale
RMSEA: root mean square error of approximation
SD: standard deviation
SEN: special educational needs
SWEWBS: Short Warwick–Edinburgh Mental Well‐being Scale
YBRSS: Youth Risk Behaviour Surveillance System

Neurodiversity, a term that originated in the autistic community in the 1990s, refers to the existence of diverse neurotypes (on a spectrum of neurotypical to neurodivergent) with varying neurocognitive profiles and needs amongst the population, with a focus of moving away from deficit‐based models and towards accepting neurodevelopmental difference, with particular focus on existing medical, educational and social structures (Botha et al. 2024; Masataka 2017; Dwyer 2022). Although neurodivergence encompasses neurodevelopmental conditions, such as autism spectrum disorders (ASD, henceforth autism), attention deficit hyperactivity disorder (ADHD) and dyslexia, it is a broader construct than a traditional diagnosis‐based model (Shah et al. 2022). This is to say that a neurodivergence approach recognises significant inter‐individual heterogeneity, where individuals may have various traits and neurocognitive differences that cannot be described by a single unifying diagnosis, and furthermore that having traits of one condition (e.g., ADHD) makes having traits of another more likely (e.g., OCD) (Lang et al. 2024; Gillberg 2010).

There is evidence to suggest that neurodevelopmental conditions are associated with an increased risk of externalising behaviours, including internally/externally directed aggression, conduct disorder and substance use (Steenfeldt‐Kristensen et al. 2020; Ding et al. 2021; Jacob et al. 2014; Wilson et al. 2013; Boyes et al. 2020; Kuja‐Halkola et al. 2015; Nikstat and Riemann 2020; Charach et al. 2011; Stickley et al. 2023) and may reflect challenges in impulse control. Of these, substance misuse, especially in children and young people, is particularly concerning as it is associated with adverse long‐term outcomes, including poorer physical and mental health, as well as detrimental social consequences such as reduced educational attainment, difficulties in interpersonal relationships, unemployment, involvement in violence and crime and increased risk of homelessness (Halladay et al. 2020; Adade et al. 2022; Alderson et al. 2017; Macleod et al. 2004). A study of 338 19‐ to 27‐year‐olds in Germany found that those with pre‐existing executive function deficits were at greater risk of escalating substance misuse (Kräplin et al. 2022). Furthermore, substance use itself may contribute to an additional deterioration in executive functioning, compounding the difficulties already commonly experienced in individuals with neurodevelopmental conditions (Kim‐Spoon et al. 2017; Brockett et al. 2018). This link of substance use and executive function deficits is particularly relevant in a neurodivergent population, where executive function deficits may contribute to neurodevelopmental phenotypes and thus may be relevant to a link between neurocognitive profiles and externalising behaviour (Safer‐Lichtenstein et al. 2024; Sadozai et al. 2024).

The existing evidence described above looks at substance use in relation to diagnosed neurodevelopmental conditions; however, there is no evidence looking at substance use in people who identify as neurodivergent. Given that the proportion of the population who identify as neurodivergent (15%–20%) is substantially higher than the population who have diagnosed neurodevelopmental conditions (c. 4%–6% for ASD and ADHD together), considering whether this broader group are also at risk of significant harm through substance misuse at a young age has important ramifications both in the short‐ and long‐term (Song et al. 2021; Centre for Disease Control and Prevention 2023; Doyle 2020), and for what preventive and treatment interventions may be best suited to their needs.

The conceptualisation of neurodivergence between a medical and social phenomenon has significant implications for how effective support and targeted interventions may be provided. Within a narrower definition of neurodevelopmental conditions with a medical model, neurodivergent people may be ‘patients’ of services (e.g., community paediatrics and child psychiatry) and therefore in receipt of medical (and hopefully holistic) care. However, those who do not fall within this narrower medical definition will not necessarily have access to such support, as it is less clear what their needs are, which evidence‐base to draw from and how healthcare input would be beneficial. Furthermore, although substance misuse is a problem for adolescents in its own right, it is associated with other issues such as poverty, familial substance misuse, adverse childhood experiences and mental health difficulties (including suicidality), where perhaps substance misuse may be an indicator of children with a broad range of other unmet needs (Karamanos et al. 2022; Hansen et al. 2025; Andrabi et al. 2017; Marino et al. 2024).

This raises questions as to whether there are unmet needs or needed interventions in this population who may well share some difficulties seen in those in neurodevelopmental services. The current best defined intervention in this instance (i.e., beyond medical care) for school‐aged children would be additional support in school, for example, in England with what is called an Education, Health and Care Plan (EHCP). EHCPs are designed to ensure effective support for learners up to the age of 25 with special educational needs (SEN). They are regulated by local authorities and based on an assessment of needs. EHCPs should incorporate a young person's views and aspirations, as well as their educational, health and social needs (Keville et al. 2025). They are, however, difficult to access for most children, and there is currently limited evidence surrounding the impact of SEN support on wider health outcomes (including substance misuse) (Zylbersztejn et al. 2023). At the time of writing, there is ongoing discussion at the government level regarding SEN reform, and in particular how support is provided outside of EHCPs (Department for Education 2026).

In summary, although it is possible to extrapolate conclusions from the evidence regarding substance misuse in ADHD/autism, populations with neurodevelopmental conditions and neurodivergent populations are overlapping but distinct. Therefore, we cannot use existing evidence to estimate the likelihood and prevalence of substance misuse in a neurodivergent population, patterns of use (e.g., particular substances used, correlates such as age or gender) or mitigating factors (e.g., appropriate support). All of these are important to those who work with neurodivergent adolescents now (e.g., education and medical professionals) and for future intervention development.

This study therefore aimed to establish whether self‐reported neurodivergence is associated with higher rates of substance misuse in children and young people. As an exploratory analysis, we examined whether EHCPs had a modulatory effect on the relationship between neurodiversity and substance misuse.

## 1 Methods

This study was conducted using data from the 2023 wave of the OxWell Student Survey (Mansfield et al. 2021). Our analysis was preregistered on the Open Science Framework (https://osf.io/3whjf), prior to access to and analysis of the data (Ward and Fazel 2024). The OxWell study has University of Oxford Research Ethics Committee approvals (Reference: R62366/RE0014).

### 1.1 Participants

The 2023 wave of the OxWell Survey included school‐aged children mainly from four areas in England—Oxfordshire, Berkshire and Milton Keynes (South East England) and Liverpool (North West England)—with a sample that broadly reflects the demographics of school‐aged children in England. Across these four areas, responses were collected from 79 secondary schools. In this analysis, we analysed data from 34 245 students in school years 7–13 (approx. ages 11–18 years). The primary school respondents were excluded as they were not asked about substance misuse.

### 1.2 Participant Cleaning

In this analysis, participants were excluded, firstly, based on a series of criteria on survey completion (incomplete consent checklist, duration of survey completion under 10 min, and incomplete demographic information) (*n* = 5738); secondly, if they had not answered the question on neurodiversity (*n* = 8803) and thirdly, if they had entered a seemingly disingenuous response to the free‐text gender question (*n* = 354) (out of concern that the rest of their data might be unreliable). We also excluded participants (*n* = 552) on age/school‐based criteria (i.e., to include only secondary school‐aged students 11‐ to 18‐year‐olds and remove older students likely attending further education colleges). We then also excluded those who did not respond to the question regarding whether they had an Educational, Health or Care Plan (EHCP) (*n* = 152). Finally, we excluded participants who responded neither yes nor no to the neurodiversity question (i.e., those that answered ‘Not Sure’ and ‘Prefer not to say’; the results of this combined group are, however, included in Supporting Information). This left a final sample size of *n* = 21 749.

### 1.3 Questionnaire

The OxWell Student Survey is a self‐report online survey, which contains up to 300 questions (depending on response patterns) and one derived variable (Index of Multiple Deprivation [IMD]). Including the 2023 version, there have been four cross‐sectional waves of survey collection. These previous waves, alongside patient and public involvement (PPI), informed the content of the 2023 survey. The survey contains a mixture of questions, which stem from both the study team (e.g., questions quantifying substance use or school‐based problems) and from pre‐existing validated questionnaires (e.g., the Short Warwick‐Edinburgh Mental Well‐being Scale (SWEMWBS), the Revised Children's Anxiety and Depression Scale (RCADS‐11)) (Mansfield et al. 2021; Shah et al. 2021; Radez et al. 2021; Fazel et al. 2021).

The survey explores the proportion of participating pupils who report experiencing mental health, behavioural and social difficulties. The survey questions are designed to ascertain this through a mixture of self‐report subjective (e.g., self‐rated attention span) and self‐report objective (e.g., frequency of substance use and frequency of school exclusion) measures.

### 1.4 Neurodiversity and SEN Variable

Participants in the survey were asked ‘Do you consider yourself to be dyslexic/dyspraxic, and/or autistic, and/or have ADHD (i.e., neurodivergent)?’ and could answer with ‘yes’, ‘no’, ‘not sure’ or ‘prefer not to say’.

Furthermore, participants were asked ‘Do you receive support for special education needs (e.g., have an EHCP: Education, Health & Care Plan)?’, with the same ‘yes’, ‘no’, ‘not sure’ or ‘prefer not to say’ options.

### 1.5 Substance Use Variables

OxWell contains data in relation to vaping, smoking, alcohol use and illicit substance use. These questions include information on frequency of use, motivations for use (vaping only) and supply of substances. In this analysis, we only examine variables that quantify substance use.

#### 1.5.1 Vaping and Smoking

There are two questions examining smoking and vaping separately in the OxWell dataset. These questions ask about the frequency of vaping/smoking with response options being: ‘Yes’ (with three qualifiers; most days, often or sometimes), ‘I used to but not in the last one month’, and ‘No’.

#### 1.5.2 Alcohol Use

Three questions on alcohol use were adapted from the Youth Risk Behaviour Surveillance System (YRBSS) (CDC 2024; Mpofu et al. 2023). Firstly, participants were asked about the frequency of alcohol intake. This was quantified by how many days in the last month they had had an alcoholic drink, with possible responses being; 0 days, 1 or 2 days, 3–5 days, 6–9 days, 10–19 days, 20–29 days or everyday.

Participants were also asked about the number of days in the past month in which they would drink four or more drinks consecutively, quantified as 0 days, 1 day, 2 days, 3–5 days, 6–9 days, 10–19 days or 20 or more days.

Finally, participants were asked about the largest number of drinks they had consumed consecutively (within a couple of hours) within the last month, rated as nil, 1 or 2 drinks, 3 drinks, 4 drinks, 5 drinks, 6 or 7 drinks, 8 or 9 drinks or 10 or more drinks.

#### 1.5.3 Recreational Drugs

Participants were asked about their use of cannabis, nitrous oxide, ketamine, ecstasy, benzodiazepines, spice and other (including amphetamines, psychedelics, fentanyl and heroin). They were asked about whether they had used these substances ever (in the last months, in the last year and more than a year ago) or never.

#### 1.5.4 Cleaning of Substance Use Variables

Firstly, nonresponses were treated as missing data and excluded from analyses. They were not assumed to indicate nonsubstance use. Missing data were not imputed.

In the interest of retaining data about substance misuse (rather than use in general), where possible and clinically relevant substances were cleaned into variables that clearly delineated this. On this principle, smoking and vaping were coded into a binary variable, based on whether participants reported their use as ‘Yes, most days’ versus any other response (‘yes, often’; ‘yes, sometimes’; ‘I used to, but not in the last one month’; ‘no’). The decision was made to use only ‘most days’ as the high use category as ‘often’ could capture significant heterogeneity between ‘most days’ and ‘sometimes’, limiting the utility of this analysis.

For alcohol variables, descriptive variables are presented across three strata of lowest, intermediate and highest use amongst the sample. For the purposes of regression analysis, these were then binarised into lowest use and highest use variables. These were number of days drinking per month (≥ 6 days vs. not); most consecutive drinks in the past month (≥ 3 drinks vs. < 3 drinks); and number of days drinking > 4 alcoholic drinks (≥ 6 days vs. < 6 days).

For illicit drug use, given that the question asked about use in the past 12 months, responses were binarised as ‘12 months used substances’ versus ‘no reported substance use history’, across all substances asked about.

### 1.6 Data Analysis

Data were analysed using *R 4.4.0*. Analyses were performed as logistic regression models, using Firth regression via the *logistf* (Rahman and Sultana 2017). This was used in order to account for zero inflation in our dataset, given the rarity of high substance use amongst our cohort.

Three iterative models were produced. The first model was an unadjusted model, comparing substance use outcomes by neurodiversity (yes vs. no). The second model was an adjusted model, which factored in demographic covariates. These covariates were gender, age, ethnicity, familial history of substance use (yes or no) and two derived variables on poverty. Our final model was the same as the previous model but also included complete total RCADS‐11 and SWEMWBS scores.

OxWell contained eight questions adapted from the Welsh Young People's Survey on Child and Family Poverty (McFarlane 2021). As these questions have not been formally validated, exploratory factor analysis was conducted using R to create factors for analysis. This was done using the *promax* rotation and maximum likelihood estimation. Using both information from Scree plots and eigenvalues, an eventual two factor loading was decided upon (with one variable excluded). The root mean square residual for this model was 0.01. The two factors were a ‘home difficulties’ variable (with five factors), as well as a ‘school difficulties’ variable (with two factors). This model was rechecked using confirmatory factor analysis, where the model had a root mean square error of approximation (RMSEA) of 0.029, and a comparative fit index of 0.992.

To account for multiple comparison, the false discovery rate correction (Benjamini–Hochberg procedure) was applied to *p*‐values (Benjamini and Hochberg 1995).

The OxWell Survey also contained one question examining motivations for vaping amongst the sample, with different items participants who vaped could endorse, as well as a free‐text response. These data were manually cleaned and descriptive statistics produced for neurotypical and neurodivergent respondents regarding common reasons for vaping.

## 2 Results

21 749 students overall were included in this analysis. The full sample description is included in Table 1. Overall, 5459 students identified as neurodivergent, which equates to 25.1% of those who responded yes or no to the question on neurodiversity, and 19.4% of those who responded at all to the neurodiversity question (including other).

**TABLE 1:** Sample description.

	Neurodivergent (*N* = 5459)	Neurotypical (*N* = 16 290)
Age: *M* (SD)	13.9 (1.9)	13.8 (1.8)
Gender*		
*Male*	2515 (46.1%)	7616 (46.8%)
*Female*	2446 (44.8%)	8339 (51.2%*)
*Other*	475 (8.7%*)	300 (1.8%)
*Missing*	23 (0.4%)	35 (0.2%)
Ethnicity*		
*White*	3448 (63.2%)*	7881 (48.4%)
*Mixed*	358 (6.6%)	874 (5.4%)
*Asian*	232 (4.2%)	3009 (18.5%)*
*Black*	134 (2.5%)	972 (6%)
*Arab*	57 (1.0%)	362 (2.2%)
*Other*	86 (1.6%)	393 (2.4%)
*Missing*	1144 (21%)	2799 (17.2%)
Family history of substance misuse		
*Yes*	524 (9.6%)	536 (3.3%)
*No*	2950 (54.0%)	11 595 (71.2%)
*Do not know*	639 (11.7%)	1095 (6.7%)
*Prefer not to say*	242 (4.4%)	366 (2.2%)
*Missing*	1104 (20.2%)	1698 (16.6%)
SWEMWS scores (mean (SD))*	18.9 (4.9)	21.5 (4.8)*
RCADS‐11 scores (mean (SD)) *	13.9 (9.1)*	13.3 (8.6)
Poverty questions *n* (%) endorsing at least 1 item	*n* (40.6%)	*n* (23.6%)

*Note:* * denotes statistically significant differences based upon Wilcoxon (numerical variables) or chi‐squared tests (categorical).

The mean sample age was 13.8 years (standard deviation [SD] 1.84), and by gender the sample had roughly equal proportions of girls (49.6%) and boys (46.6%), with 3.6% reporting their gender identity as other (gender diverse) or gender nondisclosing. When examined using chi‐squared, the gender split between neurodivergent (ND) and neurotypical (NT) groups was different; there were more gender diverse children in the ND versus NT group (8.7% vs. 1.8%) and fewer girls (44.8% vs. 51.2%).

The proportions of ethnic groups by neurodiversity were also different, with a greater proportion of White children amongst the neurodivergent group (63.2% ND vs. 48.4% NT), as well as fewer Asian children (4.2% vs. 18.5%). This difference was significant on chi‐squared testing.

There were also significant differences in mean RCADS‐11 scores (13.9 ND vs. 13.3 NT); where higher scores denote higher anxiety/depression symptoms, as well as on SWEMWS scores (18.9 ND vs. 21.5 NT; where higher scores denote better well‐being). In respect to measures of poverty, 40.6% of the neurodivergent group endorsed at least one poverty indicator, compared with 23.6% of the neurodivergent group.

Breakdown of substance use variables by year group is given in Supporting Information.

### 2.1 Main Regression Analyses

#### 2.1.1 Vaping

In our sample, 15 099 students responded to whether or not they had vaped (Tables 2, S1). Of this, overall 656 (4%) of the sample said that they were vaping on most days. This included 9% of ND children and 3% of NT children. Unadjusted logistic regression estimated the odds ratio (OR) at 3.72 (95% confidence interval (CI) 3.17–4.35, *p* < 0.0001). This effect holds when adjusting for demographic factors and for depression/anxiety and well‐being measures (ORs 2.44 and 1.91, respectively, *p*‐values < 0.0001) (Table 3).

**TABLE 2:** Smoking and vaping amongst the sample. Proportions are given of those that responded to the question (i.e., missing data excluded). Percentages are given as a percentage of respondents.

	Neurotypical	Neurodivergent
Vaping (daily)	316/11517 (3%)	340/3582 (9%)
Smoking (daily)	104/11482 (1%)	134/3568 (4%)
Drug use (past 12 months yes/no)	608/11074 (5%)	507/3394 (15%)

**TABLE 3:** Binary logistic regression analyses of vaping and smoking amongst the sample.

Model	Neurodiversity OR (95% CI)	*p*
*Vaping*		
Unadjusted	3.72 (3.17–4.35)	*p* < 0.0001
Adjusted (basic demographics)	2.24 (1.83–2.74)	*p* < 0.0001
Adjusted (demographics + RCADS/SWEMWS)	1.91 (1.53–2.38)	*p* < 0.0001
*Smoking*		
Unadjusted	4.26 (3.30–5.53)	*p* < 0.0001
Adjusted (basic demographics)	1.95 (1.38–2.75)	0.00022
Adjusted (demographics + RCADS/SWEMWS)	1.78 (1.21–2.62)	0.0042
*Drug use*		
Unadjusted	3.02 (2.67–3.42)	6E‐16
Adjusted (basic demographics)	1.92 (1.63–2.25)	1.82E‐14
Adjusted (demographics + RCADS/SWEMWS)	1.66 (1.39–1.98)	4.1E‐08

#### 2.1.2 Smoking

Overall, of 15 050 respondents, only 238 students were smoking on most days (Tables 2, S1). This included 134 (4%) of ND children and 104 (1%) of NT children. Despite an unadjusted odds ratio of 4.26 (95% CI 3.30–5.53, *p* < 0.0001), this effect was reduced although still significant when adjusting for demographic factors (95% 1.95 [1.38–2.75, *p* < 0.001]) and psychological measures (1.78 [1.21–2.62], *p* < 0.01) (Table 3).

#### 2.1.3 Alcohol

Firstly, in respect to number of days of alcohol consumption (from 14 249 overall responses), most of both groups (93% ND group and 97% NT group) reported drinking on fewer than 6 days of the month, with subsequent small differences in the numbers drinking beyond that amount (5% vs. 2% drinking 6–19 days, 2% vs. 1% 20 + days) (Table 4). Unadjusted (OR 2.67, 95% CI 2.23–3.18, *p* < 0.0001) and adjusted (OR 1.45, 95% CI 1.15–1.81, *p* < 0.05) models indicate that the neurodiverse group were more likely to drink more, although this effect became insignificant after adjusting for psychological measures (Table 5).

**TABLE 4:** Descriptive statistics of sample (given by each alcohol variable collected).

	Count	NT	ND
Number days drinking/month	< 6 Days	10 588 (97%)	3130 (93%)
	6–19 Days	227 (2%)	161 (5%)
	20+ Days	70 (1%)	73 (2%)
	Total	10 885	3364
Most consecutive drinks in past month	0–2 Drinks	9504 (90%)	2620 (80%)
	3–5 Drinks	678 (6%)	333 (10%)
	6 + Drinks	418 (4%)	318 (10%)
	Total	10 600 (76%)	3271 (24%)
Number days drinking > 4 alcoholic drinks	0–5 Days	10 228 (99%)	2954 (96%)
	6–19 Days	65 (1%)	72 (2%)
	20+ Days	42 (0.4%)	47 (2%)
	Total	10 335 (77%)	3073 (23%)

**TABLE 5:** Binomial logistic regression models of alcohol use variables.

Number of days drinking	Neurodivergence OR (95% CI)	*p*
Unadjusted	2.67 (2.23–3.18)	*p* < 0.0001
Adjusted (basic demographics)	1.45 (1.15–1.81)	0.0019
Adjusted (demographics + RCADS/SWEMWS)	1.25 (0.98–1.59)	0.077
Largest number consecutive drinks	Neurodivergence OR (95% CI)	*p*
Unadjusted (*n* = 17 744)	2.16 (1.94–2.40)	*p* < 0.0001
Adjusted (basic demographics)	1.44 (1.24–1.66)	*p* < 0.0001
Adjusted (demographics + RCADS/SWEMWS)	1.21 (1.03–1.41)	0.022
Days > 4 consecutive drinks	Neurodivergence OR (95% CI)	*p*
Unadjusted	3.90 (3.12–4.89)	*p* < 0.0001
Adjusted (basic demographics)	1.88 (1.40–2.53)	*p* < 0.0001
Adjusted (demographics + RCADS/SWEMWS)	1.62 (1.17–2.25)	0.0046

Secondly, when looking at the largest number of drinks consumed consecutively within the past month, there was a clearer difference between groups that were drinking and particularly in the highest Category of 6 or more drinks (318 (10%) of ND children, 418 (4%) of NT children) (Table 4). Firth regression demonstrated that the effect remains when controlling for basic demographic variables (aOR 1.44, 95% CI 1.24–0.166, *p* < 0.0001) and for psychological measures factors (aOR 1.21, 95% CI 1.03–1.41, *p* < 0.05) (Table 5).

Finally, when examining the number of days on which more than four alcoholic drinks were consumed consecutively, most of the sample did this on five or fewer days of the month (99% NT, 96% ND) (Table 4). However, our unadjusted analysis demonstrated that the ND group were more likely than the NT group to be in the higher use categories (OR 3.90, 95% CI 3.12–4.89, *p* < 0.0001), where this effect held after adjusting for demographic (aOR 1.88, 95% CI 1.40–2.53, *p* < 0.00001) and for psychological measures (aOR 1.62, 95% CI 1.17–2.25, *p* < 0.05) (Table 5).

#### 2.1.4 Illicit Drugs

Overall, of 14 468 responses, 1115 participants had previously used illicit drugs on at least one occasion within the past 12 months. This included 507 (15%) of ND respondents and 608 (5%) of the NT group (Table 2).

In the unadjusted model, the odds ratio was 3.02 (95% CI 2.67–3.42, *p* < 0.0001) and remained significant after controlling for demographics factors (1.92 OR, 95% CI 1.63–2.25, *p* < 0.0001) and for psychological measures (OR 1.66, 95% CI 1.39–1.98, *p* < 0.0001) (Table 3).

#### 2.1.5 Motivations for Vaping

The motivations for vaping between the ND and NT samples are given in Table S3. The most commonly endorsed reasons were similar between groups, including enjoying vaping, friends, flavours of vapes and to try vaping. From the free‐text responses, the proportion of ND students reporting vaping for mental health (e.g., stress and anxiety) was larger (7.8%) than NT students (2.0%).

### 2.2 Exploratory Analysis by EHCP

Further analyses were conducted to see whether there were differences between neurodivergent children with and without EHCPs (Tables S2, S4). For this analysis, the demographic adjusted models were used as the likely best estimate of the influence of neurodiversity. The EHCP variable was coded as a binarised variable (yes or no) and then used as an interaction term with the neurodiversity binary variable. This was done in order to preserve sample size and thus power, where a direct contrast within the neurodiverse sample would be significantly underpowered.

When rerunning basic adjusted models using this interaction term, none of the interaction terms was significant.

## 3 Discussion

This paper aimed to understand substance misuse in adolescents who self‐identify as neurodivergent. Our findings firstly demonstrate that the proportion of students who endorsed using substances (vapes/e‐cigarettes, cigarettes, alcohol and illicit drugs) regularly or at concerning levels is overall small. However, those who self‐identify as neurodivergent are more likely to be in the higher risk categories of taking substances than their nonneurodiverse peers. This relationship holds true after adjusting for demographic variables that are known to be associated with substance use, as well as after adjusting for scales of well‐being and depression/anxiety symptoms. In our exploratory analysis, we also found that students being on an EHCP did not significantly modulate the relationship between substance use and neurodivergence. These findings add to the extant literature in that they demonstrate a clear relationship between self‐identifying as neurodivergent and substance misuse, where previous studies have only looked at substance misuse within more clear diagnostic boundaries (e.g., ADHD and autism), and demonstrate that this association is independent of other highly relevant factors (e.g., familial substance misuse, poverty and intercurrent mental health difficulties).

The overall proportions of students self‐identifying with neurodivergence in this specific sample (25.1%) is much higher than the traditional neurodevelopmental conditions (ADHD and autism), which have a prevalence of around 5% in the general population (Maenner et al. 2023; Salari et al. 2023), or other conditions such as dyslexia and dyspraxia (estimated 10% and 6%, respectively) (Hulme and Snowling 2011; Cleaton et al. 2020). This would suggest that there are many respondents in our sample who identify as neurodivergent but who are not in receipt of any formalised support (medical or educational). This raises questions as to whether these students need specific support around managing substance use and how to enhance their access to care given difficulties in how they would be identified at their early stages of need, either by healthcare or education professionals, ideally preventing more functional impairment.

One potential avenue we attempted to evaluate within this study was whether having an EHCP might have some influence. However, from examining our data, it seems that only 28% of neurodivergent students reported having an EHCP (despite making up 70% of all students who were on an EHCP). Secondly, there did not seem to be a significant modulatory effect of EHCPs on the relationship between neurodiversity and substance use outcomes. This may be because of missing data in our sample (particularly amongst the ND group), inadequate coverage of EHCPs, construct validity of the questions on neurodiversity and EHCPs or that safeguarding against higher risk behaviours—such as substance misuse is beyond the scope of EHCPs (IPSEA (Independent Provider of Special Education Advice) 2024). Even if they are beyond the scope of EHCPs, one may still hypothesise there would be a protective effect of EHCPs in the model by virtue of EHCPs being hard to access, and therefore potentially signifying those with greater need or who have more involved care. In summary, these findings would suggest that an intervention beyond existing medical or educational formal support may be indicated for this population. Given planned reforms to how support can be provided outside of EHCPs to children with SEN, future work to evaluate the impact of these changes on broader health and well‐being outcomes in adolescents will be important (Department for Education 2026).

Any such intervention to help neurodivergent children with substance use would benefit from codesign with neurodivergent students, parents and teachers to help determine the best and most relevant educational and environmental approaches (Hamilton and Petty 2023; Cook 2024). This model of a more public health approach would also be in keeping with a traditional school nurse model of care or that of the Educational Mental Health Practitioners (EMHPs) that are being introduced across schools in England to provide preventive and early intervention mental health support in schools. In both of these roles that interface between health and education, safeguarding broader health and well‐being is an important part of their role (Sutton and White 2024).

Adopting universal interventions that support neurodivergent students would also remove key barriers in care and tailored support (Turuba et al. 2022; Young et al. 2021; Price et al. 2024). This could include, for example, moving away from automatic suspension or expulsion for use of illicit substances in favour of supportive approaches, as current Department of Education guidance stipulates (Department for Education 2012). From national data, the incidence of drug‐related permanent exclusions has fallen relative to overall exclusions since the pandemic (GOV.UK 2023). However, from a recent meta‐analysis, there is currently limited evidence to support the use of universal school‐based interventions in preventing substance misuse (Tinner et al. 2022). Furthermore, there is an even‐greater gap in interventions for neurodivergent children with no existing defined interventions, universal or individual, that appear sufficiently evidenced, resourced or inclusive to address this risk.

One way to potentially target might be to understand and address motivations for misuse. One motivation of interest in this population would be ‘self‐medication’, which is discussed in both the ADHD and autism literature (Weir et al. 2021; Young et al. 2023). There is also evidence to suggest that insight and diagnosis can be protective where people are taking these substances as a form of self‐medication, which may be a particular problem if access to neurodevelopmental services is limited—for example, in some UK services (Lee et al. 2017; Ward et al. 2024). From our limited data around motivations for vaping (see Supporting Information), it seemed that 7.8% of ND adolescents who vaped, and 2% of NT adolescents, explicitly reported that they vaped to alleviate mental health symptoms (and in particular stress and anxiety). The attenuated association between neurodivergence and substance use after adjusting for well‐being and anxiety/depression measures—especially in relation to number of days drinking—also lends weight to this hypothesis. Further work to characterise the subpopulations of depressed/anxious adolescents and substance misuse outcomes would be useful, as well as work to understand if ‘self‐medicators’ can be meaningfully identified from large datasets, particularly to see whether this group is distinct from neurodivergent adolescents and adolescents with significant anxiety/depression symptoms.

### 3.1 Strengths and Limitations

The main strength of this study is the large sample size and representativeness of English school‐aged children. Even with missingness of substance use variables, each substance use variable analysed in this study had at least 13 000 respondents. Furthermore, the recency of this data means that they encapsulate current trends in substance use, for example, vaping/e‐cigarette use.

A strength and limitation of this study is its broad conceptualisation of neurodivergence, which permits a far broader question to be asked than that, which could be asked if diagnosis were limited to medically diagnosed neurodevelopmental conditions. However, because specific neurodivergence's were not asked about, we cannot establish whether the association with substance misuse is specific to one or more groups, for example, ADHD where impulsiveness is more prominent than in some other neurodivergent groups (e.g., dyslexia). Further research is needed to explore substance use in relation to specific strands of neurodivergence, but more broadly to understand the nature of the relationship between having a neurodevelopmental condition versus identifying as neurodivergent and how this might relate to the needs of adolescents.

The main limitation of this study would be the accuracy of self‐report data. Although actions were taken to remove individuals who may have unreliable data (e.g., those who entered free‐text genders that were likely disingenuous), it is not possible to say whether substance use data are accurate. However, this is a limitation of all self‐report and self‐report substance use data, and objective measures of substance use would be unfeasible for a number of reasons, including the scale of testing needed, the resource implications of testing and the nonidentifiable nature of the survey.

Additionally, we excluded the ‘prefer not to say’ and ‘not sure’ groups in our analyses of neurodiversity and of EHCPs. This may introduce a form of non‐response bias, where there may be reasons leading to their reluctance to disclose their identity that may relate to their wider behaviour.

## 4 Conclusion

This study aimed to understand whether those who self‐identify as neurodivergent were more at risk of substance misuse than their peers. The findings suggest that, although the overall number of students who may be using substances at high levels is small, neurodivergent young people are three times more likely to be in this group than their neurotypical peers. In the context of the relatively large proportion of neurodivergent students, scalable universal approaches to substance misuse that incorporate neurodiversity‐affirming principles need to be developed and evaluated. This study highlights the need to better understand neurodiverse young people outside of discrete neurodevelopmental conditions and to focus on how educational settings can potentially be a key location for preventative interventions as well as facilitating access to necessary early support.

## Author Contributions

**John Headley Ward:** methodology, writing – review and editing, writing – original draft, formal analysis, conceptualization. **Francesca Happé:** writing – review and editing, supervision. **Mina Fazel:** conceptualization, investigation, writing – original draft, funding acquisition, writing – review and editing, methodology, supervision.

## Funding

This research was funded by the NIHR Applied Research Collaboration Oxford and Thames Valley at Oxford Health NHS Foundation Trust (M.F), and the Westminster Foundation with support from Liverpool City Council, Milton Keynes City Council, NHS Frimley ICB, NHS Buckinghamshire, Oxfordshire and Berkshire West ICB, and Oxfordshire City Council. F.H. is part‐funded by the National Institute for Health and Care Research (NIHR) Maudsley Biomedical Research Centre and King's College London (KCL).

The views expressed in this publication are those of the authors and not necessarily those of the National Institute for Health and Care Research or the Department of Health and Social Care.

J.H.W. is funded by an NIHR Academic Clinical Fellowship.

## Ethics Statement

The OxWell study has University of Oxford Medical Sciences Interdivisional Research Ethics Committee approvals (Reference: R62366/RE0014, 19 January 2023). Informed consent of participants over 16, and informed participant assent for under 16 s was taken, with the option for parents to opt out on a child's behalf.

## Consent

Informed consent of participants over 16, and informed participant assent for under 16 s was taken, with the option for parents to opt out on a child's behalf.

## Conflicts of Interest

The authors declare no conflicts of interest.

## Supporting information




**Figure S1:** (a + b) Vaping amongst neurodivergent and neurotypical adolescents, subdivided by year group.
**Figure S2:** (a + b) Smoking amongst neurodivergent and neurotypical adolescents, subdivided by year group.
**Figure S3:** (a + b) Number of days having > 4 consecutive drinks amongst neurodivergent and neurotypical adolescents, subdivided by year group.
**Figure S4:** (a + b) Number of days drinking amongst neurodivergent and neurotypical adolescents, subdivided by year group.
**Figure S5:** (a + b) Most consecutive drinks amongst neurodivergent and neurotypical adolescents, subdivided by year group.
**Figure S6:** (a + b) Number of days having > 4 consecutive drinks amongst neurodivergent and neurotypical adolescents, subdivided by year group.
**Table S1:** Smoking and vaping data disaggregated.
**Table S2:** Table denoting proportions of NT vs. ND students with an EHCP in place.
**Table S3:** Data on reasons for vaping, by neurodivergence (% given as a proportion of respondents who had ever vaped). Other includes responses with an < 1% endorsement.
**Table S4:** cch70357‐sup‐0001‐Supplementary_Material.pdf. *p*‐values of interaction term of neurodiversity × EHCPs in the adjusted regression models.

## Data Availability

Data are available on request from the authors.
