# Supplementary material for: Are School‐Aged Children Who Self‐Identify as Neurodivergent at Greater Risk of Substance Misuse? A Study of 20 000 Secondary School Students
Source: Child Care Health Dev. 2026 Sep 24;52(5):e70357. doi: 10.1111/cch.70357 (PMC13603042; doi:10.1111/cch.70357)
Supplement: Supplementary file 1 [file CCH-52-e70357-s001.pdf]

**Supplementary Figures**

**Figure S1a+b:** vaping amongst neurodivergent and neurotypical adolescents, subdivided by year group.

S1a (Neurodivergent)

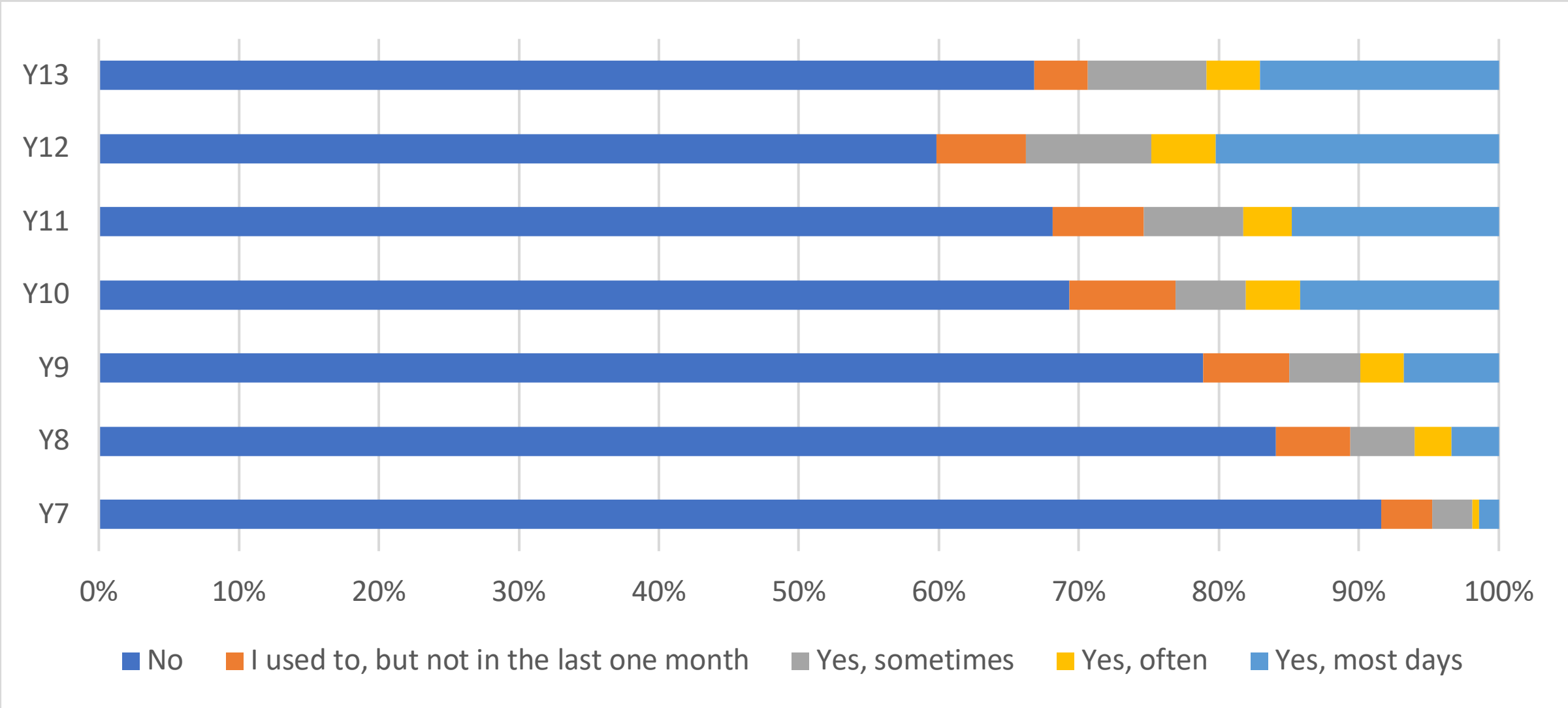


S1b (Neurotypical)

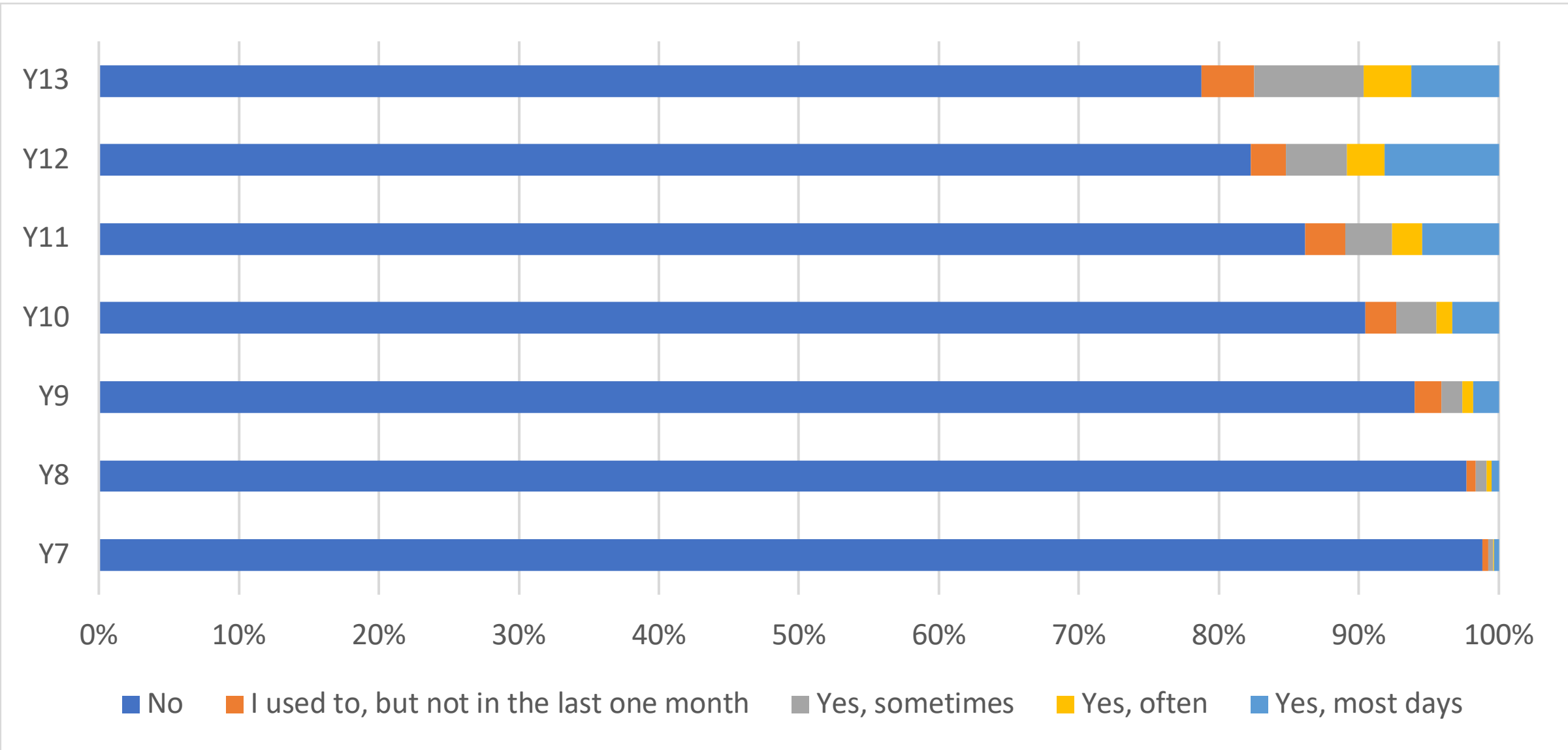

**Figure S2a+b:** smoking amongst neurodivergent and neurotypical adolescents, subdivided by year group.

S2a (Neurodivergent)

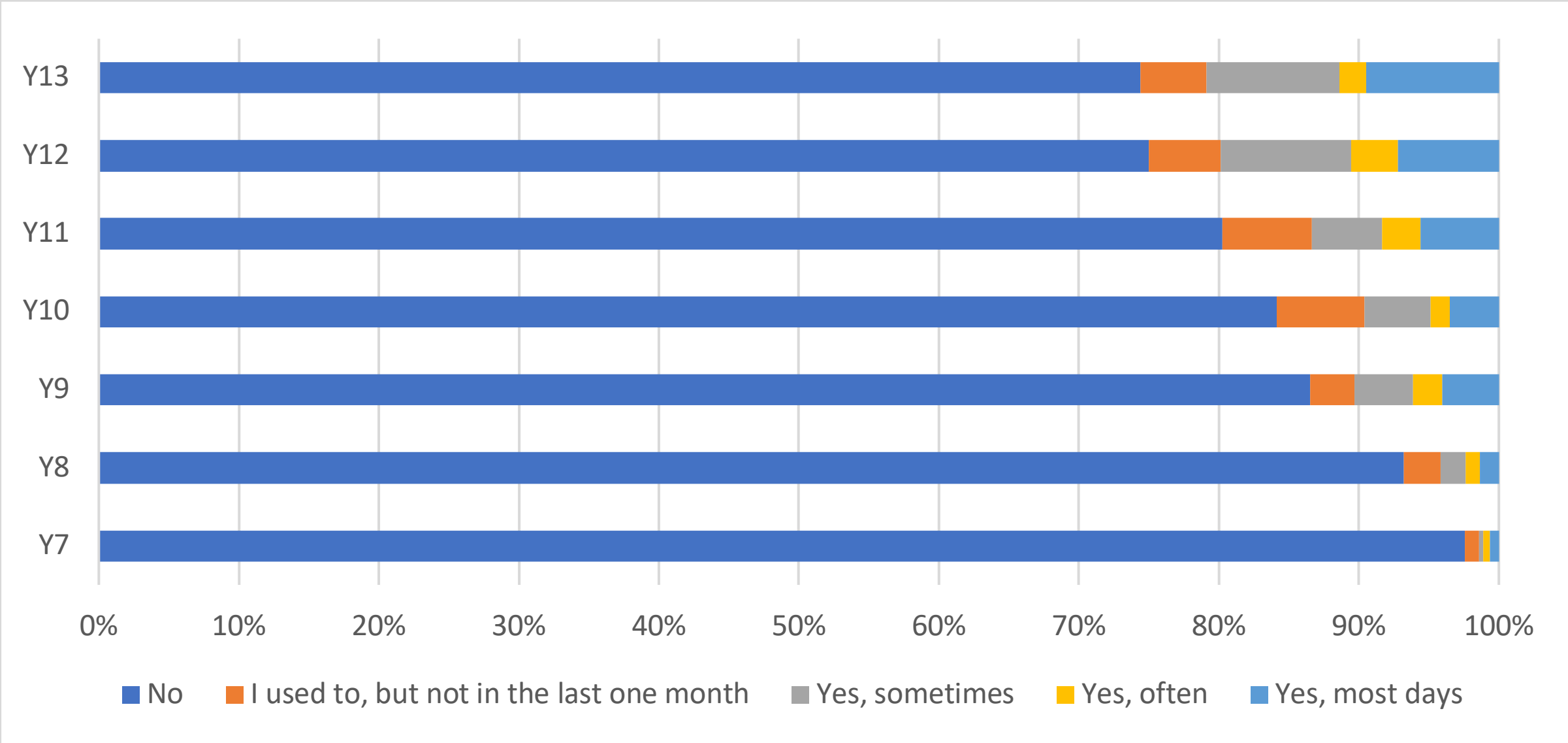


S2b (Neurotypical)

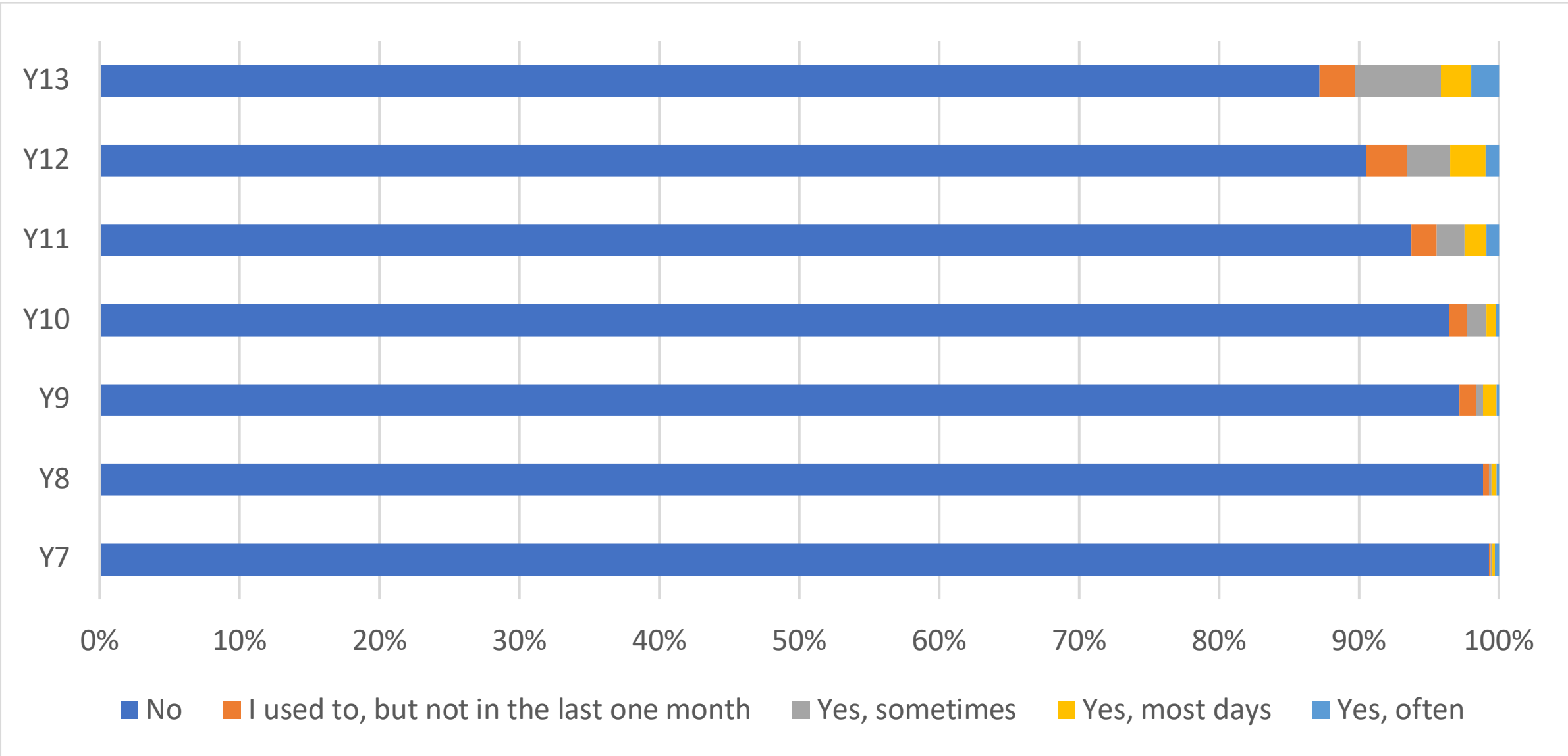

**Figure S3a+b:** Number of days having >4 consecutive drinks amongst neurodivergent and neurotypical adolescents, subdivided by year group.

S3a (Neurodivergent)

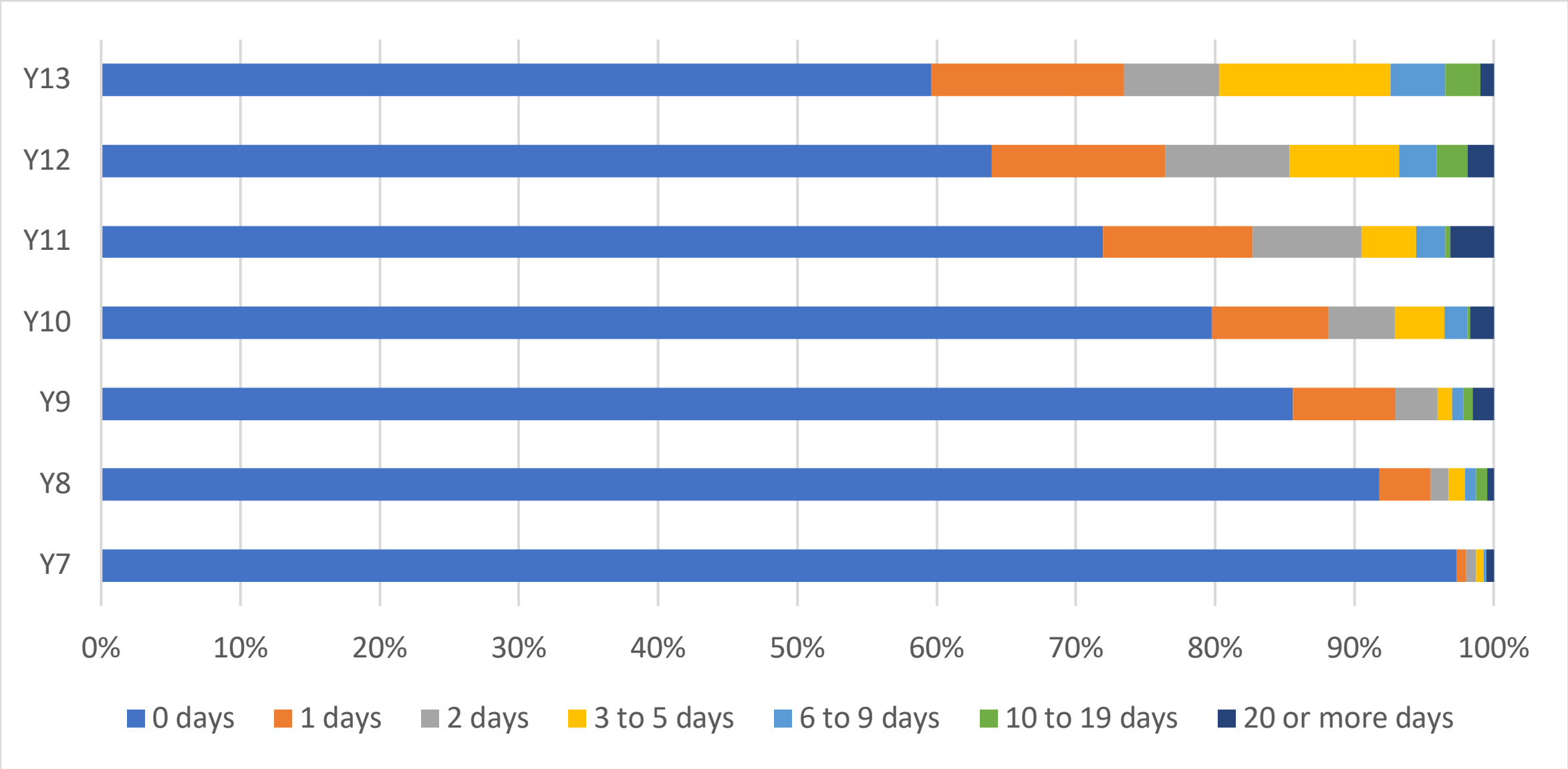


S3b (Neurotypical)

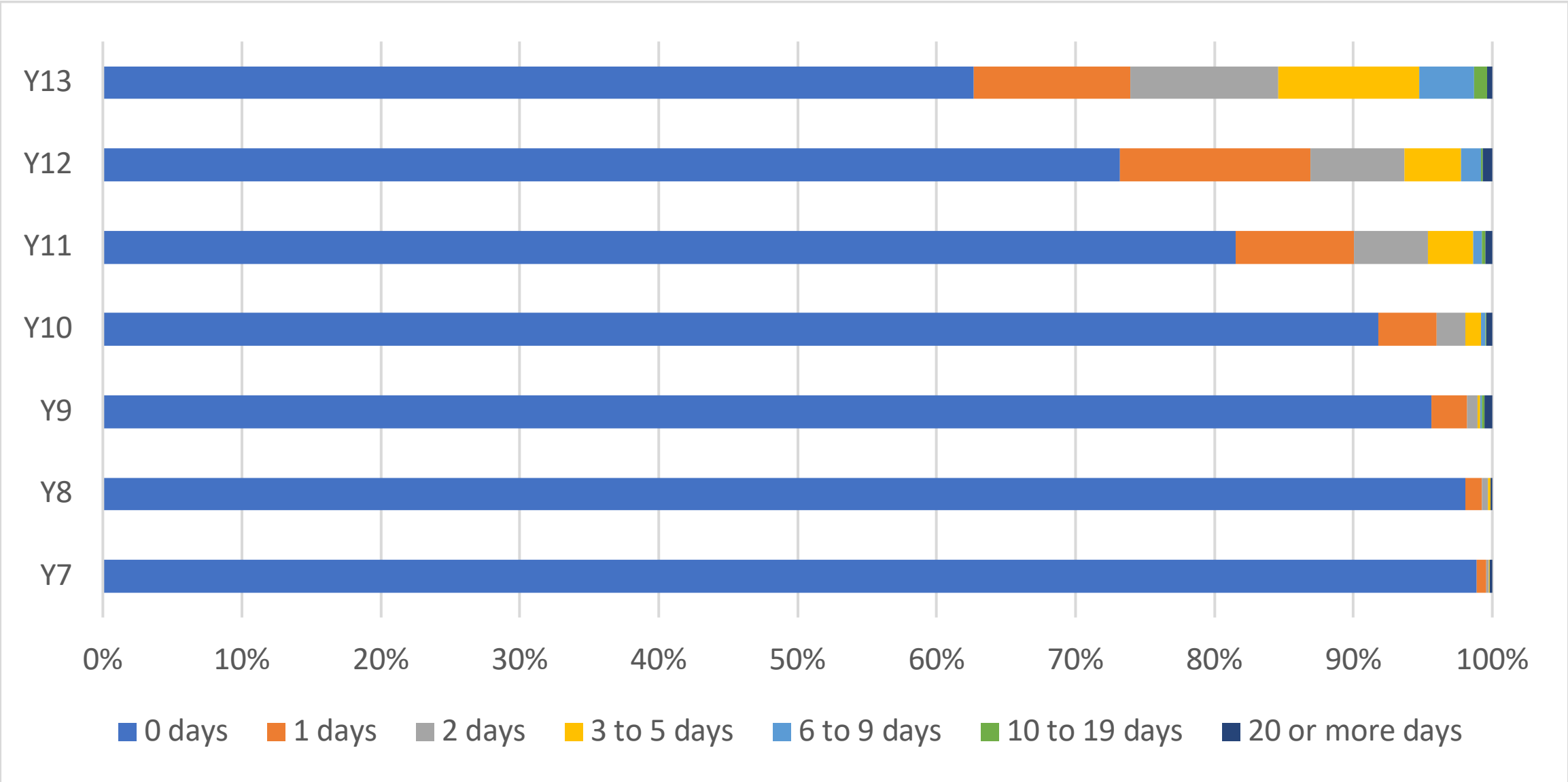

**Figure S4a+b:** Number of days drinking amongst neurodivergent and neurotypical adolescents, subdivided by year group.

S4a (Neurodivergent)

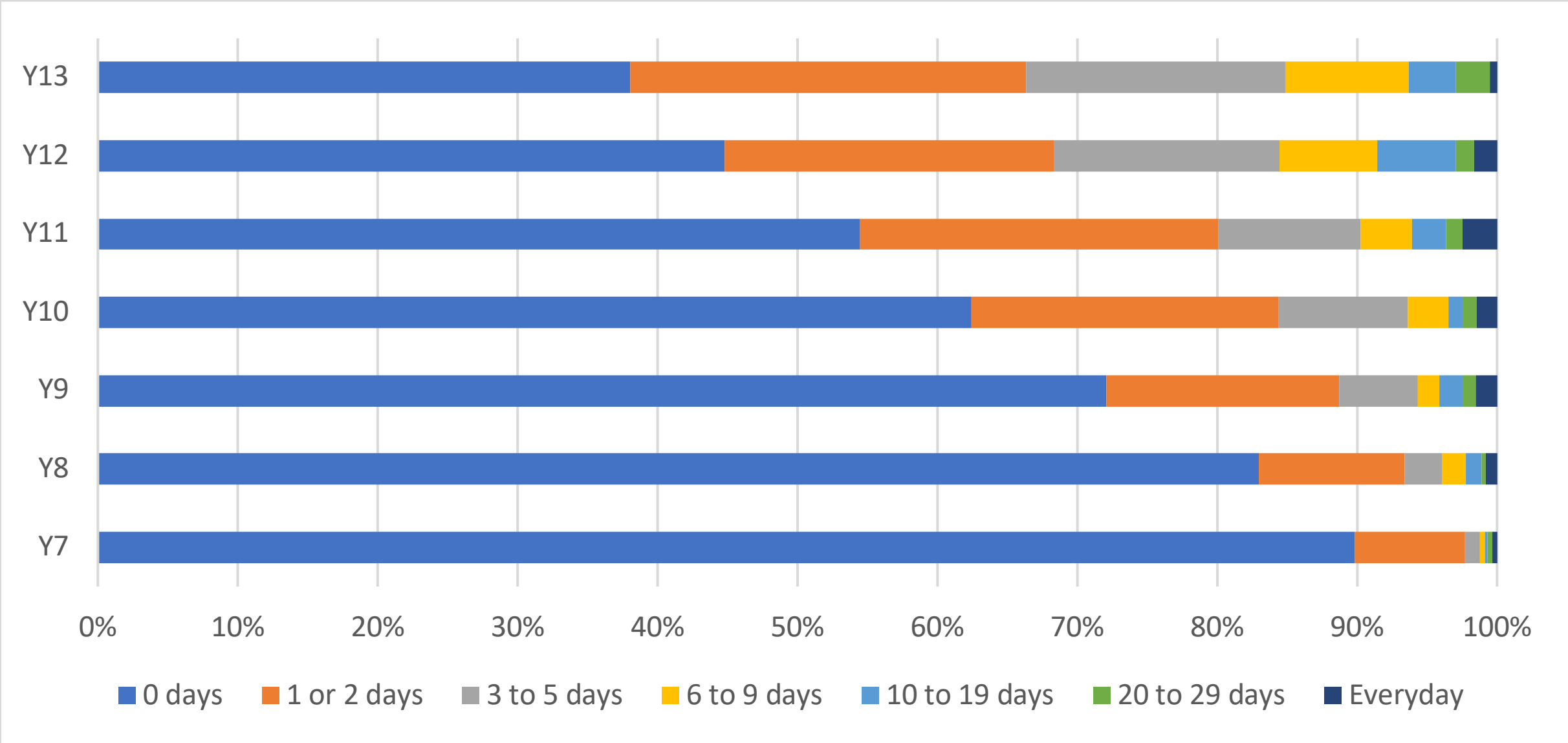


S4b (Neurotypical)

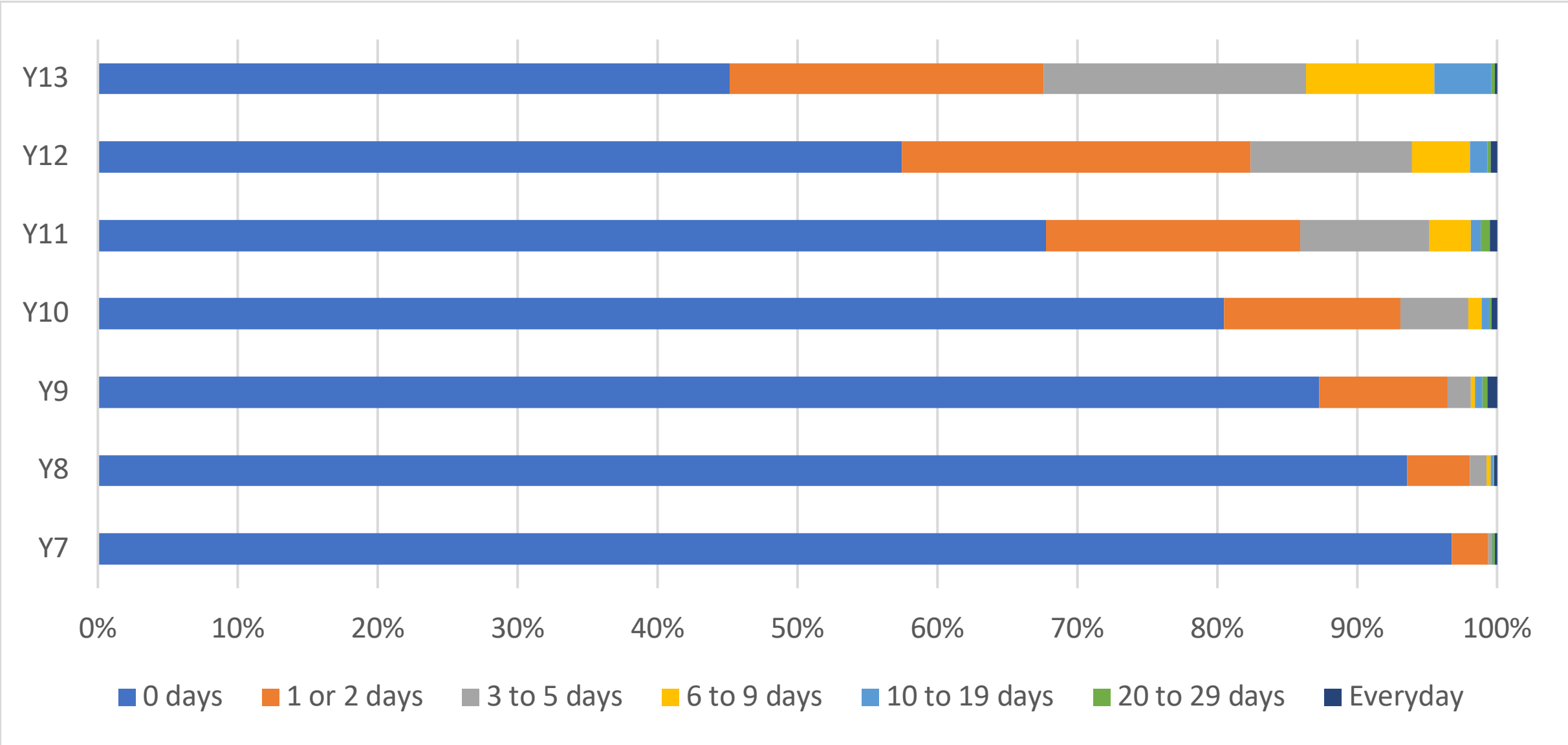

**Figure S5a+b:** Most consecutive drinks amongst neurodivergent and neurotypical adolescents, subdivided by year group.

S5a (Neurodivergent)

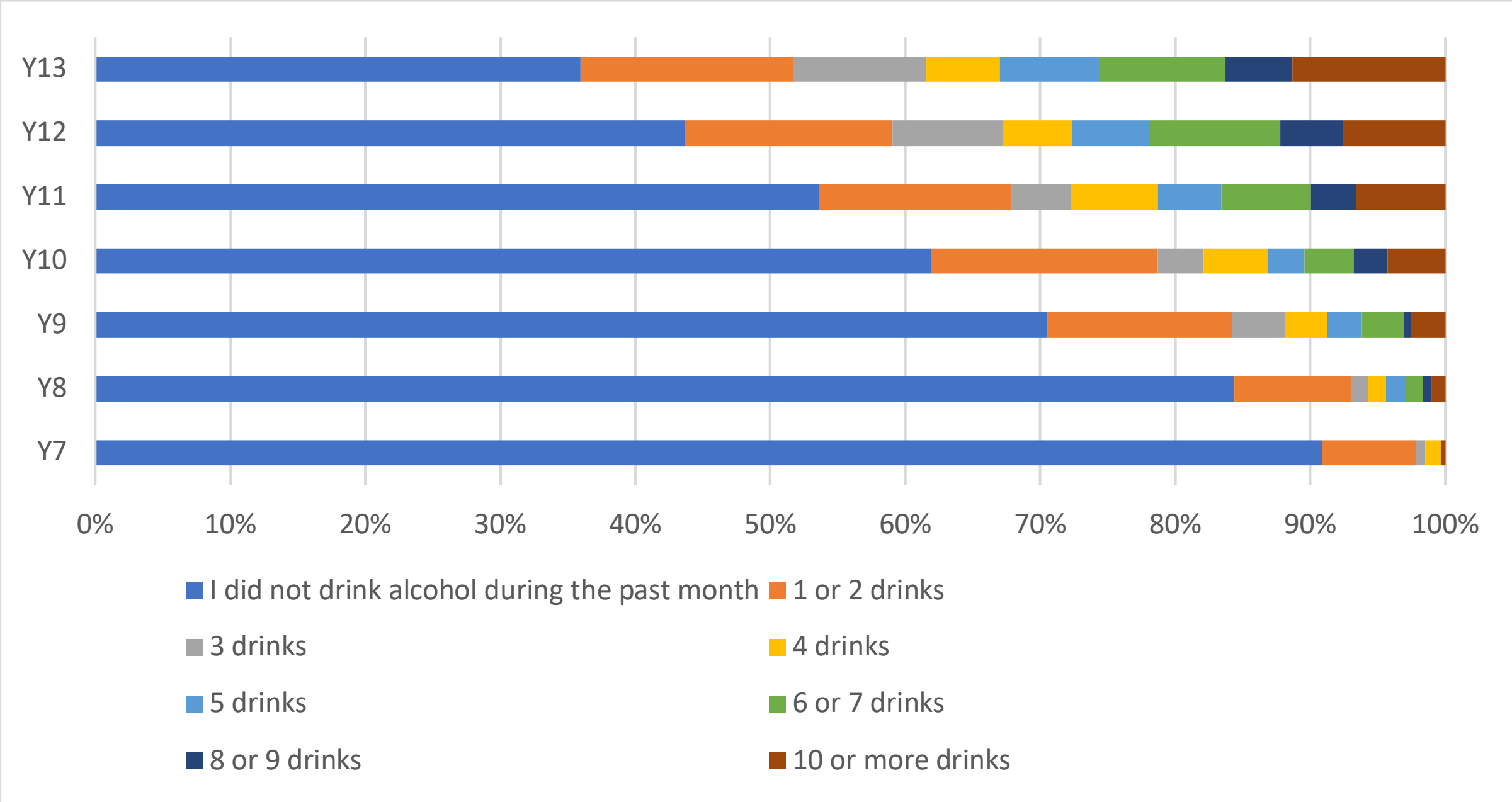


S5b (Neurotypical)

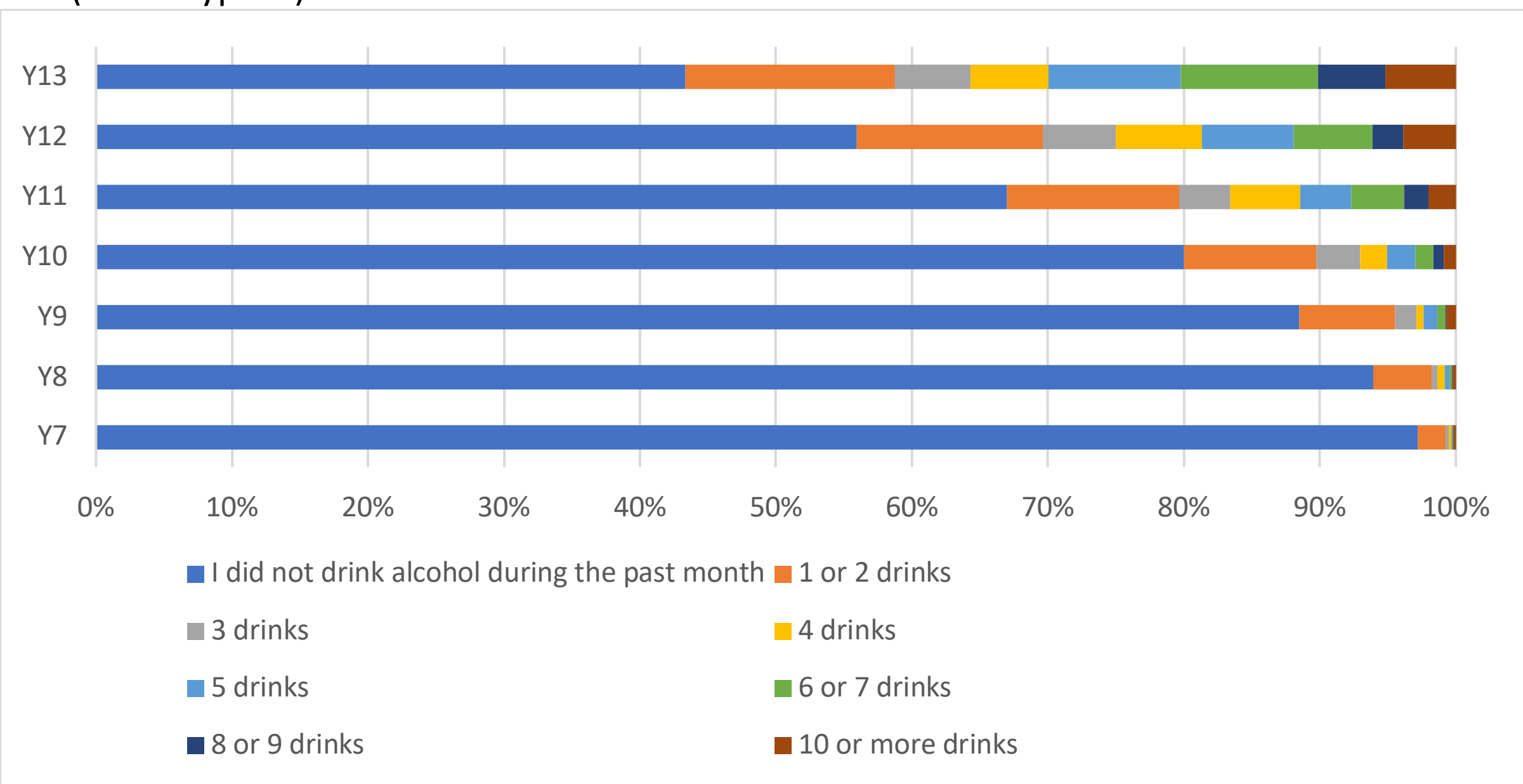

**Figure S6a+b:** Number of days having >4 consecutive drinks amongst neurodivergent and neurotypical adolescents, subdivided by year group.

S6a (Neurodivergent)

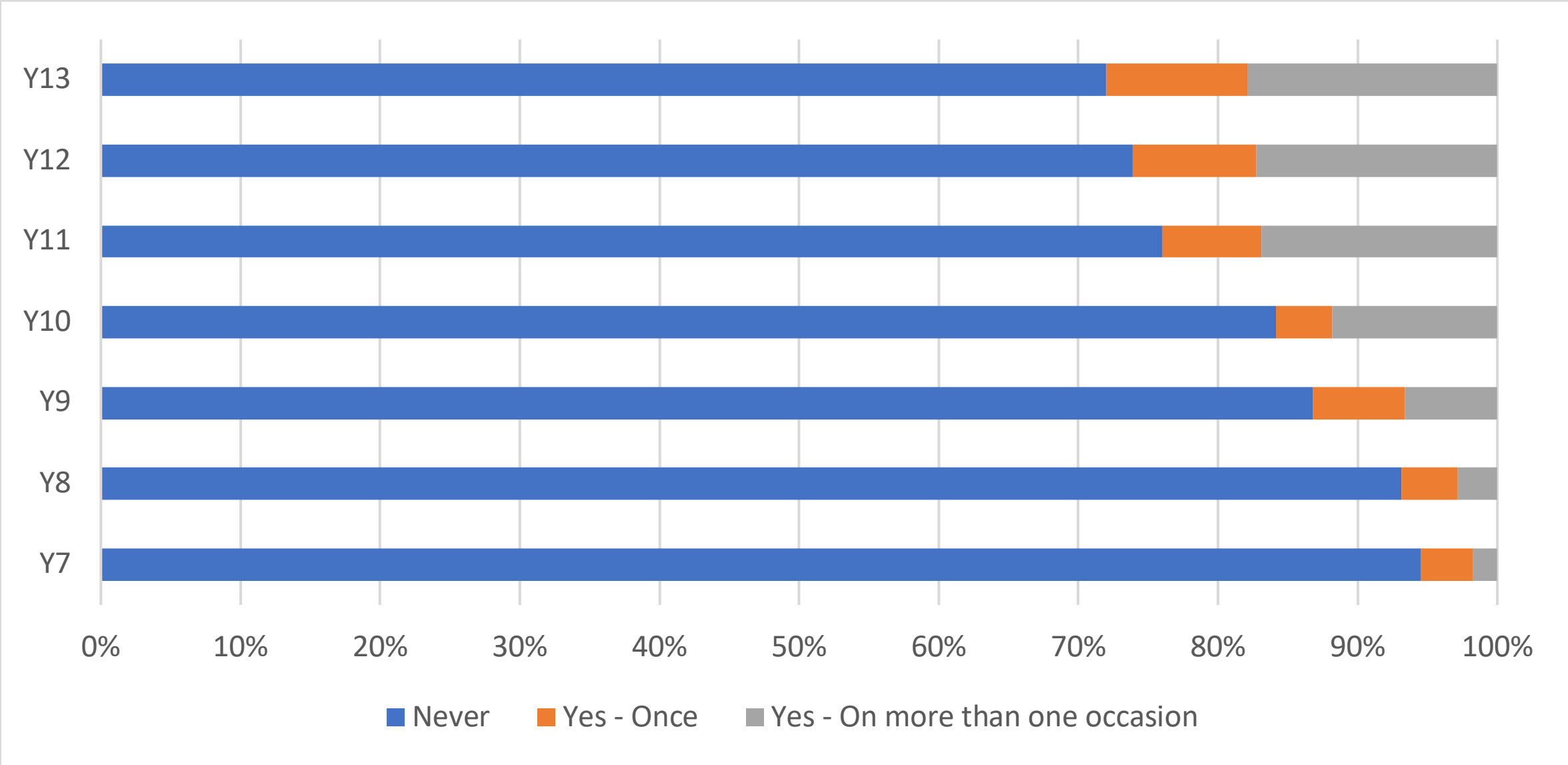


S6b (Neurotypical)

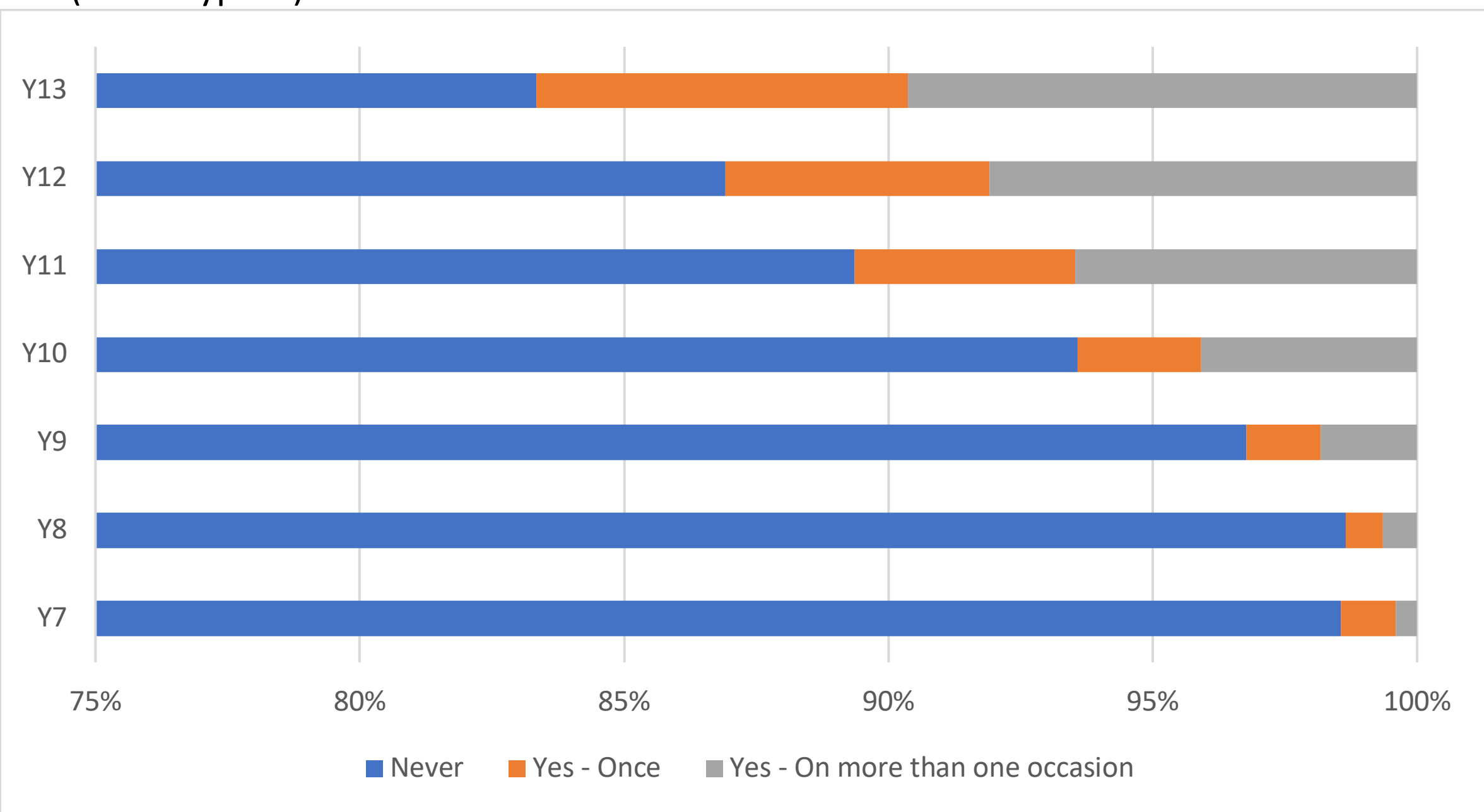

## Supplementary Information

Table S1: smoking and vaping data disaggregated

| Smoking? | Neurodivergent | | Total |
|---|---|---|---|
| | **No** | **Yes** | |
| **Yes, most days** | 104 (0.64%) | 134 (2.45%) | 238 (1.09%) |
| **Yes, often** | 53 (0.33%) | 61 (1.12%) | 114 (0.52%) |
| **Yes, sometimes** | 136 (0.83%) | 147 (2.69%) | 283 (1.30%) |
| **I used to, but not in the last one month** | 133 (0.82%) | 139 (2.55%) | 272 (1.25%) |
| **No** | 11056 (67.87%) | 3087 (56.55%) | 14143 (65.03%) |
| **No Response** | 4808 (29.52%) | 1891 (34.64%) | 6699 (30.80%) |
| **Total** | 16290 (74.90%) | 5459 (25.10%) | 21749 (100.00%) |

| Vaping? | Neurodivergent | | Total |
|---|---|---|---|
| | **No** | **Yes** | |
| **Yes, most days** | 316 (1.94%) | 340 (6.23%) | 656 (3.02%) |
| **Yes, often** | 126 (0.77%) | 105 (1.92%) | 231 (1.06%) |
| **Yes, sometimes** | 242 (1.49%) | 197 (3.61%) | 439 (2.02%) |
| **I used to, but not in the last one month** | 195 (1.20%) | 204 (3.74%) | 399 (1.83%) |
| **No** | 10638 (65.30%) | 2736 (50.12%) | 13374 (61.49%) |
| **No Response** | 4773 (29.30%) | 1877 (34.38%) | 6650 (30.58%) |
| **Total** | 16290 (74.90%) | 5459 (25.10%) | 21749 (100.00%) |

Table S2: table denoting proportions of NT vs ND students with an EHCP in place

| EHCP In Place | Neurodiversity | |
|---|---|---|
| | Neurotypical | Neurodivergent |
| No | 14781 (91%) | 2778 (51%) |
| Yes | 650 (4%) | 1528 (28%) |
| Missing | 859 (5%) | 1153 (21%) |
| Total | 16290 (100%) | 5459 (100%) |

Table S3: data on reasons for vaping, by neurodivergence (% given as a proportion of respondents who had ever vaped). Other includes responses with an <1% endorsement.

| **Motivation to Vape** | **Neurodivergent** | |
|---|---|---|
| | No | Yes |
| **To stop using tobacco** | 6.6% | 7.3% |
| **To reduce tobacco, but not stop completely** | 5.8% | 7.1% |
| **To stay off tobacco** | 4.6% | 6.6% |
| **As something to help in situations where I can't smoke** | 10.6% | 14.4% |
| **To not put others at risk of second-hand smoke** | 4.1% | 6.9% |
| **To save money** | 6.5% | 8.4% |
| **Feel that they are addicted to tobacco** | 6.8% | 10.5% |
| **Enjoy vaping** | 40.8% | 37.9% |
| **Advised by healthcare professional** | 3.4% | 3.0% |
| **Advised by a friend** | 21.5% | 19.1% |
| **To try it** | 47.2% | 37.6% |
| **Because of the flavours** | 30.9% | 28.3% |
| **Because of friends** | 43.7% | 36.1% |
| **Nonsense Response** | 0.3% | 1.1% |
| **Mental Health** | 2.0% | 7.8% |
| **No response** | 0.7% | 0.9% |
| **Other** | 1.1% | 1.3% |

Table S4: p values of interaction term of neurodiversity x EHCPs in the adjusted regression models.

| **Adjusted Model** | **P Value** |
|---|---|
| **Vaping** | 0.14 |
| **Smoking** | 0.55 |
| **Number of Days Drinking Consecutively** | 0.85 |
| **Number of days drinking >4 alcoholic drinks** | 0.53 |
| **Most consecutive drinks** | 0.98 |
| **Drug use** | 0.72 |
